# Supplementary material for: Integration of absolute multi-omics reveals dynamic protein-to-RNA ratios and metabolic interplay within mixed-domain microbiomes
Source: Nat Commun. 2020 Sep 18;11:4708. doi: 10.1038/s41467-020-18543-0 (PMC7501288; doi:10.1038/s41467-020-18543-0)
Supplement: Supplementary file 1 — Supplementary Information [file 41467_2020_18543_MOESM1_ESM.pdf]

SUPPLEMENTARY INFORMATION

Integration of absolute multi-omics reveals dynamic protein-to-RNA ratios and metabolic interplay within mixed-domain microbiomes.

Delogu et al.

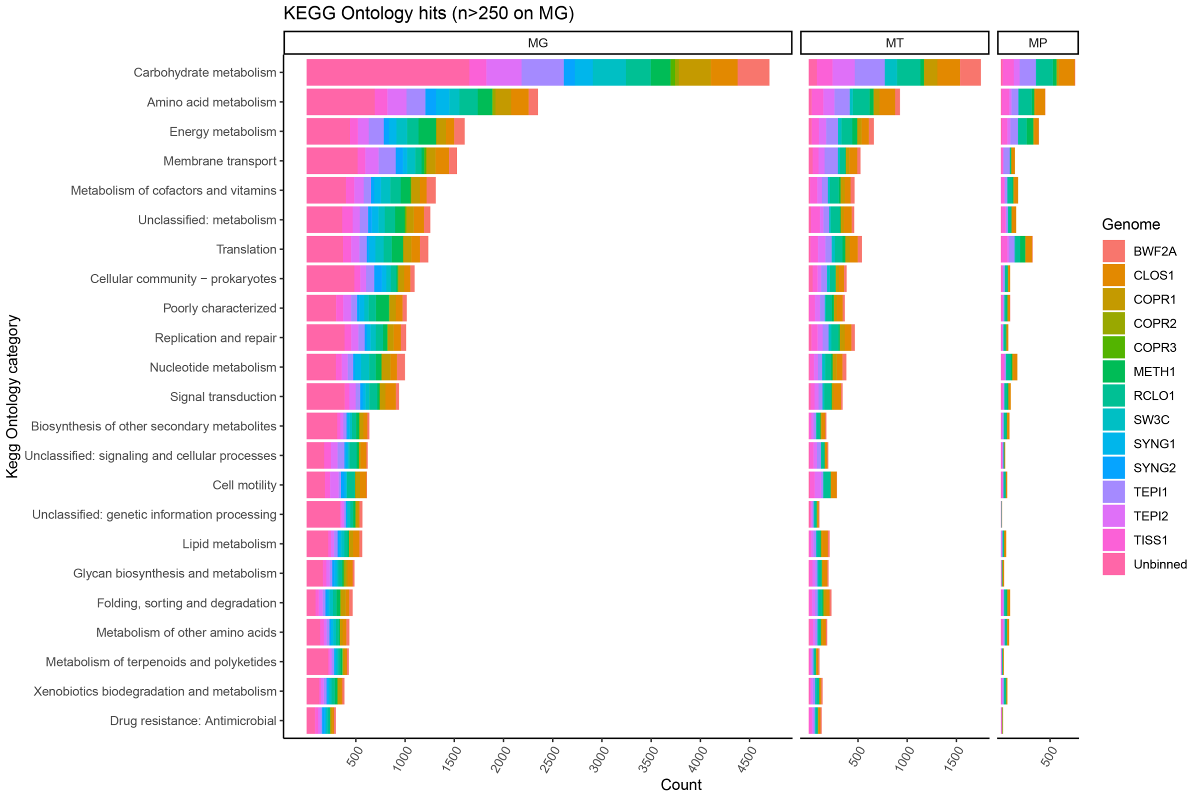

Supplementary Figure 1. Total counts of KEGG Orthology hits within the different omic layers per KEGG category. The colors represent the metagenomic bins reconstructed from the SEM1b consortium to which the annotated ORFs belong. The sorting of the KEGG categories was performed using the counts for the metagenome (MG, hence relative to the genomic potential), and it is more conserved in the metatranscriptome (MT, two-sided Kendall tau: 0.77, p=5.3×10^{9} not adjusted) than in the metaproteome (MP, two-sided tau 0.68, p=5.5×10^{6} not adjusted).

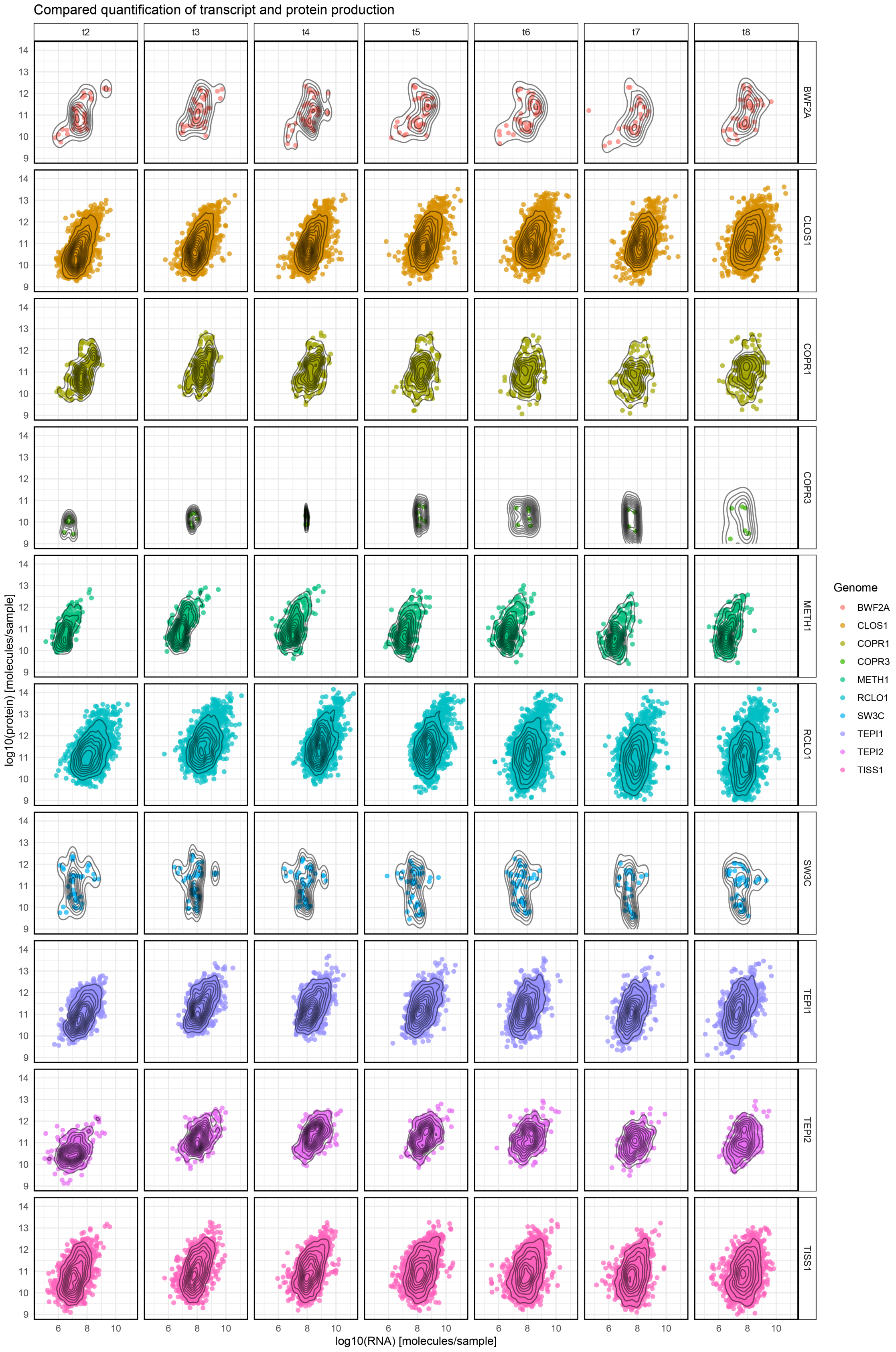

Supplementary Figure 2. Per-time-point scatterplots of the absolute protein and transcript levels for ORFs that produced both detectable transcript and protein in SEM1b MAGs.
