# Supplementary material for: Integration of absolute multi-omics reveals dynamic protein-to-RNA ratios and metabolic interplay within mixed-domain microbiomes
Source: Nat Commun. 2020 Sep 18;11:4708. doi: 10.1038/s41467-020-18543-0 (PMC7501288; doi:10.1038/s41467-020-18543-0)
Supplement: Supplementary file 3 — Description of Additional Supplementary Files [file 41467_2020_18543_MOESM3_ESM.docx]

**Description of Supplementary Files**

**File Name: Supplementary Data 1**

**Description:** Catalogue of the annotated SEM1b ORFs used in this study, including ORF-, contig- and MAG-identifiers.

**File Name: Supplementary Data 2**

**Description:** Catalogue of ORF identifiers and their annotated KEGG Orthology code.

**File Name: Supplementary Data 3**

**Description:** Filtered expression table for the SEM1b metatranscriptome (MT), measures in log10(molecules+1). Each column corresponds to an ORF identifier, and each row to a sample timepoint (t2-8) and replicate (A-C).

**File Name: Supplementary Data 4**

**Description:** Filtered expression table for the SEM1b metaproteome (MP), measured as log10(molecules+1). Each column corresponds to an ORF identifier, and each row to a sample timepoint (t2-8) and replicate (A-C).

**File Name: Supplementary Data 5**

**Description:** The main output from the PECA-R analysis. Each row represents an ORF identifier, the columns called R and D contain the estimate of the 35 translation and protein degradation rates respectively. The CPS columns contain the 36 score for being a change point in the parameters and the FDR columns store the False 37 Discovery Rate associated to the CPS.X

**File Name: Supplementary Data 6**

**Description:** MT read pairs summary per sample. The columns indicate the quantified set of read pairs. “Starting” the whole set of raw read pairs in the beginning of the analysis. “Filtered”: the read pairs remaining after preprocessing and filtering. “Filtered (fraction)”: the fraction of filtered read pairs respect to the starting read pairs. “Filtered (biological)”: filtered biological read pairs (i.e. without the internal standard). “Filtered (biological, fraction)”: fraction of biological filtered read pairs respect to the filtered ones. “Filtered (internal standard)”: the read pairs isolated from the internal standard. “Mapped”: read pairs mapped on the ORF collection. “Mapped (frac)”: fraction of read pairs mapped on the ORF collection respect to the “Filtered (biological)” set.

**File Name: Supplementary Data 7**

**Description:** Repartition of crude RPMK values per sample per bin. The rows represent the samples (as indicated in the first column). The columns from the second to the thirty-fourth one represent the sum of RPMK values of the ORFs from the given bin in the given sample. Those columns alternate raw RPMK values and the fraction represented in the sample. The last column contains the sample RPMK total sum.
