# Supplementary material for: Integration of absolute multi-omics reveals dynamic protein-to-RNA ratios and metabolic interplay within mixed-domain microbiomes
Source: Nat Commun. 2020 Sep 18;11:4708. doi: 10.1038/s41467-020-18543-0 (PMC7501288; doi:10.1038/s41467-020-18543-0)
Supplement: Supplementary file 11 — Reporting Summary [file 41467_2020_18543_MOESM11_ESM.pdf]

nature research

Corresponding author(s): Phil B. Pope
Francesco Delogu

Last updated by author(s): Aug 21, 2020

Reporting Summary

Nature Research wishes to improve the reproducibility of the work that we publish. This form provides structure for consistency and transparency in reporting. For further information on Nature Research policies, see Authors & Referees and the Editorial Policy Checklist .

Statistics

For all statistical analyses, confirm that the following items are present in the figure legend, table legend, main text, or Methods section.

n/a	Confirmed	
☐	☒	The exact sample size (*n*) for each experimental group/condition, given as a discrete number and unit of measurement
☐	☒	A statement on whether measurements were taken from distinct samples or whether the same sample was measured repeatedly
☐	☒	The statistical test(s) used AND whether they are one- or two-sided<br>*Only common tests should be described solely by name; describe more complex techniques in the Methods section.*
☒	☐	A description of all covariates tested
☒	☐	A description of any assumptions or corrections, such as tests of normality and adjustment for multiple comparisons
☐	☒	A full description of the statistical parameters including central tendency (e.g. means) or other basic estimates (e.g. regression coefficient) AND variation (e.g. standard deviation) or associated estimates of uncertainty (e.g. confidence intervals)
☐	☒	For null hypothesis testing, the test statistic (e.g. *F*, *t*, *r*) with confidence intervals, effect sizes, degrees of freedom and *P* value noted<br>*Give P values as exact values whenever suitable.*
☒	☐	For Bayesian analysis, information on the choice of priors and Markov chain Monte Carlo settings
☒	☐	For hierarchical and complex designs, identification of the appropriate level for tests and full reporting of outcomes
☐	☒	Estimates of effect sizes (e.g. Cohen's *d*, Pearson's *r*), indicating how they were calculated

Our web collection on statistics for biologists contains articles on many of the points above.

Software and code

Policy information about availability of computer code

Data collection	The metagenomic reads were trimmed and filtered with cutadapt v2.7 and fastx v0.0.14, mapped against the two isolate strains using BWA-MEM v0.7.17-r1188, the remaining ones were assembled with Metaspades v3.10.0 and binned with Metabat v0.26.3. The ORFs and their functions were predicted using the Integrated Microbial Genomes and Microbiomes system (pipeline v4.15.1). The metatranscriptomics reads were trimmed and filtered using Trimmomatic v.0.36; rRNA and tRNA were removed and the pGEM-3Z reads extracted using SortMeRNA v.2.1b. RNA expression was quantified using kallisto v.0.41.1 and mmseq v.1.0.9. The metaproteomics raw files were processed with MaxQuant v.1.4.1.2 and the MaxLFQ algorithm to quantify the LFQ levels. The protein sysntesis and degradation rates and changing points were computed with PECAplus_cmd_line (https://github.com/PECAplus/PECAplus_cmd_line).
Data analysis	The whole analysis is broken down in R Markdown notebooks and stored at https://github.com/fdelogu/SEM1b-Multiomics. In detail R v.3.5.3 and the following data analysis package: MetQy_1.1.0 was used.

For manuscripts utilizing custom algorithms or software that are central to the research but not yet described in published literature, software must be made available to editors/reviewers. We strongly encourage code deposition in a community repository (e.g. GitHub). See the Nature Research guidelines for submitting code & software for further information.

Data

Policy information about availability of data

All manuscripts must include a data availability statement. This statement should provide the following information, where applicable:

- Accession codes, unique identifiers, or web links for publicly available datasets
- A list of figures that have associated raw data
- A description of any restrictions on data availability

All sequencing reads have been deposited in the sequence read archive (SRP134228) and all microbial genomes are publicly available on JGI under the analysis project numbers listed in Supplementary Table 6 from the paper "From proteins to polysaccharides: lifestyle and genetic evolution of Coprothermobacter proteolyticus". The mass spectrometry proteomics data have been deposited to the ProteomeXchange Consortium via the PRIDE partner repository with the dataset identifier PXD016242.

Field-specific reporting

Please select the one below that is the best fit for your research. If you are not sure, read the appropriate sections before making your selection.

☒ Life sciences ☐ Behavioural & social sciences ☐ Ecological, evolutionary & environmental sciences

For a reference copy of the document with all sections, see nature.com/documents/nr-reporting-summary-flat.pdf

Life sciences study design

All studies must disclose on these points even when the disclosure is negative.

Sample size	Sample size not relevant for the study, we used all the samples and all the genes available.
Data exclusions	Already at the bench level, some differences could be observed during sampling for t7C. Cellulose degradation was more advanced than for the other replicates and the gas concentration and composition was different, with notably more methane being present in the bottle. The findings were supported by the preliminary data exploration with PCA and hierarchical clustering, indicating that T7C was not representative of its time point. The decision was already taken in a previous work on the SEM1b community (PMID: 30315317)
Replication	Every time point in the time series was produced in triplicate. Replicates were highly similar (protein quantification yelded an average R2=0.85), exluded t7C.
Randomization	Not relevant as it was an exploratory study assessing microbial community dynamics.
Blinding	Not relevant as it was an exploratory study assessing microbial community dynamics.

Reporting for specific materials, systems and methods

We require information from authors about some types of materials, experimental systems and methods used in many studies. Here, indicate whether each material, system or method listed is relevant to your study. If you are not sure if a list item applies to your research, read the appropriate section before selecting a response.

Materials & experimental systems

n/a	Involved in the study
☒	☐ Antibodies
☒	☐ Eukaryotic cell lines
☒	☐ Palaeontology
☐	☒ Animals and other organisms
☒	☐ Human research participants
☒	☐ Clinical data

Methods

n/a	Involved in the study
☒	☐ ChIP-seq
☒	☐ Flow cytometry
☒	☐ MRI-based neuroimaging

Animals and other organisms

Policy information about studies involving animals; ARRIVE guidelines recommended for reporting animal research

Laboratory animals	*For laboratory animals, report species, strain, sex and age OR state that the study did not involve laboratory animals.*
Wild animals	*Provide details on animals observed in or captured in the field; report species, sex and age where possible. Describe how animals were caught and transported and what happened to captive animals after the study (if killed, explain why and describe method; if released, say where and when) OR state that the study did not involve wild animals.*

Field-collected samples	*For laboratory work with field-collected samples, describe all relevant parameters such as housing, maintenance, temperature, photoperiod and end-of-experiment protocol OR state that the study did not involve samples collected from the field.*
Ethics oversight	*Identify the organization(s) that approved or provided guidance on the study protocol, OR state that no ethical approval or guidance was required and explain why not.*

Note that full information on the approval of the study protocol must also be provided in the manuscript.
